# Maternal choices and preferences for screening strategies of gestational diabetes mellitus: A exploratory study using discrete choice experiment

**DOI:** 10.3389/fpubh.2022.864482

**Published:** 2022-11-01

**Authors:** Tingting Xu, Yan Jiang, Xiuyan Guo, Julie A. Campbell, Hasnat Ahmad, Qing Xia, Xiaozhen Lai, Di Yan, Liangkun Ma, Hai Fang, Andrew J. Palmer

**Affiliations:** ^1^Department of Health Management and Policy, School of Public Health, Capital Medical University, Beijing, China; ^2^China Center for Health Development Studies, Peking University, Beijing, China; ^3^Obstetrics and Gynecology, Dong E Hospital, Liaocheng, China; ^4^Menzies Institute for Medical Research, University of Tasmania, Hobart, TAS, Australia; ^5^Department of Public Health Sciences, University of Rochester Medical Center, New York, NY, United States; ^6^Obstetrics and Gynecology, Peking Union Medical College Hospital, Beijing, China

**Keywords:** discrete choice experiment, gestational diabetes mellitus, patient preferences, screening methods, Chinese pregnant women

## Abstract

**Aims:**

This study aimed to investigate maternal preferences for gestational diabetes mellitus (GDM) screening options in rural China to identify an optimal GDM screening strategy.

**Methods:**

Pregnant women at 24–28 gestational weeks were recruited from Shandong province, China. A discrete choice experiment (DCE) was conducted to elicit pregnant women's preferences for GDM screening strategy defined by five attributes: number of blood draws, out-of-pocket costs, screening waiting-time, number of hospital visits, and positive diagnosis rate. A mixed logistic model was employed to quantify maternal preferences, and to estimate the relative importance of included attributes in determining pregnant women's preferences for two routinely applied screening strategies (“one-step”: 75 g oral glucose tolerance test [OGTT] and “two-step”: 50 g glucose challenge-test plus 75 g OGTT). Preference heterogeneity was also investigated.

**Results:**

*N* = 287 participants completed the DCE survey. All five predefined attributes were associated with pregnant women's preferences. Diagnostic rate was the most influential attribute (17.5 vs. 8.0%, OR: 2.89; 95%CI: 2.10 to 3.96). When changes of the attributes of “two-step” to “one-step” strategies, women's uptake probability from full “two-step” to “one-step” significantly increased with 71.3% (95%CI: 52.2 to 90.1%), but no significant difference with the first step of “two-step” (−31.0%, 95%CI: −70.2 to 8.1%).

**Conclusion:**

Chinese pregnant women preferred the “one-step” screening strategy to the full “two-step” strategy, but were indifferent between “one-step” and the first step of “two-step” strategies.

## Introduction

Gestational Diabetes Mellitus (GDM) is a condition in which women without previously diagnosed overt diabetes exhibit high glucose intolerance during pregnancy, particularly during their third trimester (1). It has become an increasingly serious public health problem both in China and worldwide (1–3). In 2019, the overall prevalence of GDM was estimated at 14.8% of pregnant women in China (3), and 14.5% in rural China (4). GDM affected over two million pregnant women in China each year, with half of these women residing in rural areas (3–5). The occurrence of hyperglycemia in pregnancy is associated with worse (short-term and long-term) health outcomes for mothers as well as their offspring (6). A series of epidemiological studies indicated that women with GDM had higher risk of pre-eclampsia, premature birth, macrosomia, and type 2 diabetes after childbirth (7, 8). Their babies were also at greater risk of obesity, diabetes and metabolic syndromes later in life (9, 10).

GDM screening and subsequent treatment and management are critical for women with GDM at 24–28 weeks of gestation (1, 11). Despite a number of attempts to determine an optimal and uniform screening strategy for GDM (e.g., exploring the clinical and economic effectiveness) (12), no national consensus on the best practices and criteria for GDM screening and diagnosis exists (13). Currently, “one-step” and “two-step” are the two strategies that are commonly implemented in China and other counties. For the “one-step” strategy, a 75 g oral glucose tolerance test (OGTT) is performed to a fasting woman. Then, fasting, 1 and 2 h glucose level is measured, and the recommended diagnostic threshold is 5.1, 10, and 8.5 mmol/L, respectively. Pregnant women with any single abnormal glucose value are classified as diagnosed with GDM. While for “two-step,” 50 g glucose challenge test (GCT) is firstly conducted to pregnant women (first step); if the 1-h glucose level is >7.8 mmol/L, the 75 g OGTT is then conducted to this woman next day (second step).

Some organizations including the American Diabetes Association (ADA) (1), the International Federation of Gynecology and Obstetrics (FIGO) (14) and World Health Organization (WHO) (15) recommended “one-step” strategy for women at 24–28 weeks of gestation since the diagnostic cut-off is much lower than that of the first step of the “two-step” strategy (namely 50 g GCT), which could avoid missed diagnoses (that could be also explained having higher specificity but reducing its sensitivity) and potential adverse events of hyperglycemia according to the Hyperglycemia and Adverse Pregnancy Outcomes Study (HAPO) (16). However, other international organizations such as the American College of Obstetricians and Gynecologists (17), Society of Obstetricians and Gynecologists of Canada (18), and the National Institutes of Health (19) do not support the superiority of the “one-step” over “two-step” strategy due to inadequate supporting evidence. For example, the lower cut-off value of the “one-step” strategy could result in misdiagnosis and increased risk of maternal and neonatal complications due to over-intervention and emotional stress (13, 20, 21). Furthermore, “one step” strategy asks subjects to visit hospital only one time, while “two-step” strategy might need them twice if tested positive in the first stage, which brings challenges to women living far away from a hospital. Generally, the number of blood draws of the “one-step” strategy is higher than the “two step” strategy (considering around 50% pregnant women do not need to receive the second step of “two-step” strategy). Correspondingly, the “one-step” strategy is generally costly than the “two-step.” However, if women need to experience the entire two steps, they pay more than those who only experienced the first step of the “two-step” strategy. Therefore, the two strategies come with their own advantages and disadvantages.

The inconsistent criteria of GDM caused a big challenge for pregnant women (13, 22), and brought difficulties to the promotion of GDM screening and subsequent management, especially in rural China (13, 22) where the lower GDM screening acceptance and compliance exist (23, 24). Achieving a uniform strategy of GDM is of uppermost priority. Maternal preferences on GDM screening provide us with a new direction of thinking, a more favored strategy could be conducive to improve screening acceptance, compliance and uptake (25). However, most studies in this field have focused on the differences in effectiveness of various screening strategies from the clinical perspective (13, 20), none have explored preferences and choices of screening criteria from the pregnant woman's perspective. Therefore, our present study aimed to investigate pregnant women's preferences for GDM screening to identify their preferred screening option. The findings from this study can be helpful in efficient resource allocation and healthcare decision-making processes on GDM screening in China.

## Materials and methods

### Validated guidelines and ethics approval

This study was registered in the Chinese Clinical Trial Registry (registration number: ChiCTR-DOD-16009246; http://www.chictr.org.cn/index.aspx). It was conducted in accordance with the STROBE (Strengthening the Reporting of Observational Studies in Epidemiology) Statement for reporting observational studies.

Ethics approvals were obtained from the Ethics Committee of Peking Union Medical College Hospital, Chinese Academy of Medical Sciences (Approval Number: ZS-1119).

### Study setting and sampling

In present China, the screening strategy of two steps was mainly applied in rural areas. Therefore, this study was conducted in a county hospital in Shandong province of China, where two GDM screening strategies are in practice. The per capita income in this county was ~13,242 Chinese Yuan [CNY] in 2018, which is similar to the average income in 2018 (14,600 CNY) in Chinese rural areas (26, 27). Eligible women were identified from the hospital's obstetric and gynecological outpatient department between 1st November 2016 and 31st January 2017. Women meeting the following study inclusion criteria were considered for the study: (1) clinically presenting at 24–28 weeks of gestation; (2) without overt diabetes before pregnancy (i.e., type 1 diabetes and type 2 diabetes); (3) pregnant with a single fetus; and (4) without severe comorbidities such as hypertension, renal disease, thalassemia, systemic lupus erythematosus, coeliac disease, thyroid disease and physical, or cognitive disability.

We computed a minimum sample size according to Johnson and Orme's formula: *N* ≥ 500^*^c/ (t^*^a), in which *t* indicates the number of choice tasks, *a* indicates the number of alternatives, and *c* indicates the largest number of levels for any of the attributes (28–30). We also perform a *post hoc* analysis to show the changes of a sufficient sample range when the power of test changed from 0.80 to 0.90, and odds ratio of attributes from 0.1 to 4.0 with α level of 0.05. Trained nurses contacted participants, obtained their written informed consent, and arranged the first appointment.

### Discrete choice experiment (DCE)

We investigated pregnant women's preferences for GDM screening using DCE, a commonly adopted stated preference technique (31–33). We hypothesized that the uptake of GDM screening strategies can be described by a set of attributes (e.g., diagnostic rate, number of blood draws). A series of choice tasks was developed to compare pairs of screening profiles featured by predefined multilevel attributes. For each choice task, participants were required to select a screening profile that they preferred to the other profile(s). Based on their repeated choices, the relative preferences for different attributes and levels were estimated.

#### Attributes and levels

The initial selection of attributes was informed by the literature review (13, 34), pilot individual interviews of pregnant women, and expert interviews (obstetrics and gynecology specialists, endocrinologists, nutritionists, and public health professionals). Five attributes for the DCE were: (1) the number of blood draws (1, 3, and 4); (2) out-of-pocket costs (CNY10, CNY 30, CNY 60 and CNY 90 [1 CNY = 0.145 US Dollar on January 2020]); (3) screening waiting-time (0.5, 2, and 2.5 h); (4) the number of hospital visits (1 and 2); and (5) diagnostic rate (this attribute indicates GDM positive diagnosis rate [8.0, 10.5, and 17.5%]) (Table 1). The diagnostic rate was identified from the literature review (13, 34), and the number of blood draws, screening waiting-time, out-of-pocket costs and the number of hospital visits were identified through the pilot interviews and expert consultations.

**Table 1:** Attributes and levels.

**Attribute**	**Level**	**Conceptual definitions**
Number of blood draws	One blood draw Three blood draws Four blood draws	The total number of blood draws of completing GDM screening per pregnant woman
Screening waiting-time	0.5 h 2.0 h 2.5 h	Waiting time from arriving at the outpatient departments to completing GDM screening per pregnant woman
Out-of-pocket costs^#^	10 CNY 30 CNY 60 CNY 90 CNY	Out-of-pocket costs for GDM screening
Number of hospital visits	One hospital visit Two hospital visits	The total number of hospital visits of completing GDM screening per pregnant woman
Diagnostic rate	8.0% 10.5% 17.5%	The positive rate of pregnant women defined with GDM

^#^1 Chinese Yuan (CNY) = USD 0.145 on January 2020.

#### Experimental design

With five attributes at two to four levels, a total of 216 (3^3^ × 4 × 2) hypothetical screening profiles were produced, and 46,656 (216 × 216) choice tasks containing two screening profiles were generated. NGene was used to select a subset of these possible choice tasks with a D-efficient fractional factorial experimental design (35). The D-efficient approach retains optimal orthogonality in a fractional design, and reduces the number of necessary combinations relative to a full orthogonal design. We generated 16 screening options. An example of a DCE choice task is shown in Supplements 1, 2. Accordingly, 14 choice tasks were constructed, and divided into two survey blocks (36, 37). Respondents were randomly assigned to one of the two survey blocks that contained seven choice tasks (37). This study was designed as a forced-choice study, and participants were not allowed to opt-out. Any participants who missed one question of the choice tasks were excluded from this analysis. Further details were revealed in the questionnaires (Supplement Questionnaire).

### Questionnaire development and testing

A pilot test was conducted with ten women to test the feasibility of the questionnaire. None of the participants reported any problems with the pilot test, after which the format and wording of the pilot version was refined, and the finalized version was temporarily administered by trained nurses.

The final questionnaire consisted of two sections: general characteristics (including socio-demographics [e.g., maternal age, living areas (rural areas: county and county below [county below included villages and towns]), parity [delivery times: 0 = primipara, 1 = multipara], education, household income and occupation]) and DCE section (comprising of seven choice tasks). We also set a testing question to verify the DCE result on women's preferred choice for “one-step” and “two-step” (Supplement Questionnaires).

The survey commenced with training for participants which included an introduction to the study and predefined multilevel GDM screening attributes. The meaning of diagnosis rate was explained as the positive rate, and the advantages and disadvantages of diagnosis rate was also emphasized in this training.

### Statistical analysis

Participants' characteristics were presented as Numbers (N) and percentages (%) for categorical variables, and means with standard deviations (SD) for continuous data. Statistical analyses were performed in STATA version 17 (Stata Corp LP, College Station, TX, USA). Detailed description of the statistical methods was showed in Supplement 3.

Discrete choice data was analyzed using the panel mixed logistic (PML) models with maximum simulated likelihood estimation which accommodated the nature of the data (38). As each respondent completed 7 choice tasks, and that included 14 answers (also be explained 14 samples), these answers (samples) may be correlated. The PML model extends the standard conditional logistic model by allowing one or more of the parameters in the model to be randomly distributed and the coefficients in the model to vary across respondents. It also accounts for preference heterogeneity between respondents, i.e., respondents are allowed to have different preferences, and adjust the standard errors of utility estimates to account for repeated choices by the same individual.

In the analyses, all attributes were specified as random coefficients, and choice scenarios were identified using a grouping variable. Then a higher-level grouping was specified at the level of the respondent to account for multiple choice scenarios per respondent and to account for preference heterogeneity (39).

The theoretical model describing the utility of screening profiles was based on the attributes as follows:

U = ${\hat{\beta }}_{0}$ $+{\hat{\beta }}_{1}$ ^*^(3 blood draws) + ${\hat{\beta }}_{2}$ ^*^(4 blood draws) + ${\hat{\beta }}_{3}$ ^*^(CNY 30 out-of-pocket costs) + ${\hat{\beta }}_{4}$ ^*^(CNY 60 out-of-pocket costs) + ${\hat{\beta }}_{5}$ ^*^(CNY 90 out-of-pocket costs) + ${\hat{\beta }}_{6}$ ^*^(2 hours screening waiting-time) + ${\hat{\beta }}_{7}$ ^*^(2.5 hours screening waiting-time) + ${\hat{\beta }}_{8}$ ^*^(2 hospital visits) + ${\hat{\beta }}_{9}$ ^*^(10.5% diagnostic rate) + ${\hat{\beta }}_{10}$ ^*^(17.5% diagnostic rate) + ${\hat{\beta }}_{11}^{*}$attributes^*^individual characteristics + ε

U describes the utility of a specific screening profile based on the attributes that were included in the DCE. The dependent variable represents whether a particular screening profile was chosen. The independent variables are the attribute levels that made up the screening profile (40). ${\hat{\beta }}_{0}$ represents the alternative specific constant, ${\hat{\beta }}_{1}$ to ${\hat{\beta }}_{10}$ are the attribute estimates that indicated the relative importance of each attribute. Difference in coefficients (as preference weights) between the most and least favorable levels of an attribute was interpreted as the relative importance of this attribute.

In this DCE model, women's characteristics were covariates. Therefore, we also assumed that individual characteristics, such as living areas, parity, education level and household income, yielded differing interaction effects on attributes (Supplement 4). ${\hat{\beta }}_{11}$ is the estimate for the interaction between attributes and the individual characteristics.

We further estimated the marginal probabilities when one of attributes changed from lower level to higher one and other attributes were defaulted at mean values or set at specified values. The method and mechanism of changes of probabilities calculation referred WHO's DCE guidelines (37). The formula is based on regression coefficient ($\hat{\beta }$) of DCE (37):


$$\begin{array}{l} P_{i}=\frac{e^{\beta^{{}^{\prime }}x_{i}}}{\sum^{\beta^{{}^{\prime }}x_{j}}} \end{array}$$

Where P_i_ indicates the changes of uptake probability from a screening profile j to another screening profile i. The changes of uptake probabilities of women with different characteristics were estimated as well.

The uptake probabilities for pregnant women from the least favorable attributes (8% diagnostic rate, CNY 90 out-of-pocket costs, four blood draws, two hospital visits, and 2.5 h screening waiting-time) to the most favorable attributes (17.5% diagnostic rate, CNY 10 out-of-pocket costs, one blood draw, one hospital visit, and 0.5-h screening waiting-time) were estimated. We separately optimized each attribute, and kept the remaining attributes at the least favorable (reference) levels to calculate the changes of uptake probabilities compared with the least favorable option.

We estimated the changes of women's uptake probabilities (37, 41) for the “one-step” strategy (with attributes of 17.5% diagnostic rate, CNY 30 out-of-pocket costs, three blood draws, one hospital visit, and 2-h screening waiting-time) from the first step of “two-step” strategy (with attributes of 8% diagnostic rate, CNY 10 out-of-pocket costs, one blood draw, one hospital visit, and 0.5-h screening waiting-time); and from the entire “two-step” strategy (with attributes of 8% diagnostic rate, CNY 60 out-of-pocket costs, four blood draws, two hospital visits, and 2.5-h screening waiting-time).

## Results

### Participant characteristics

**Figure 1:**
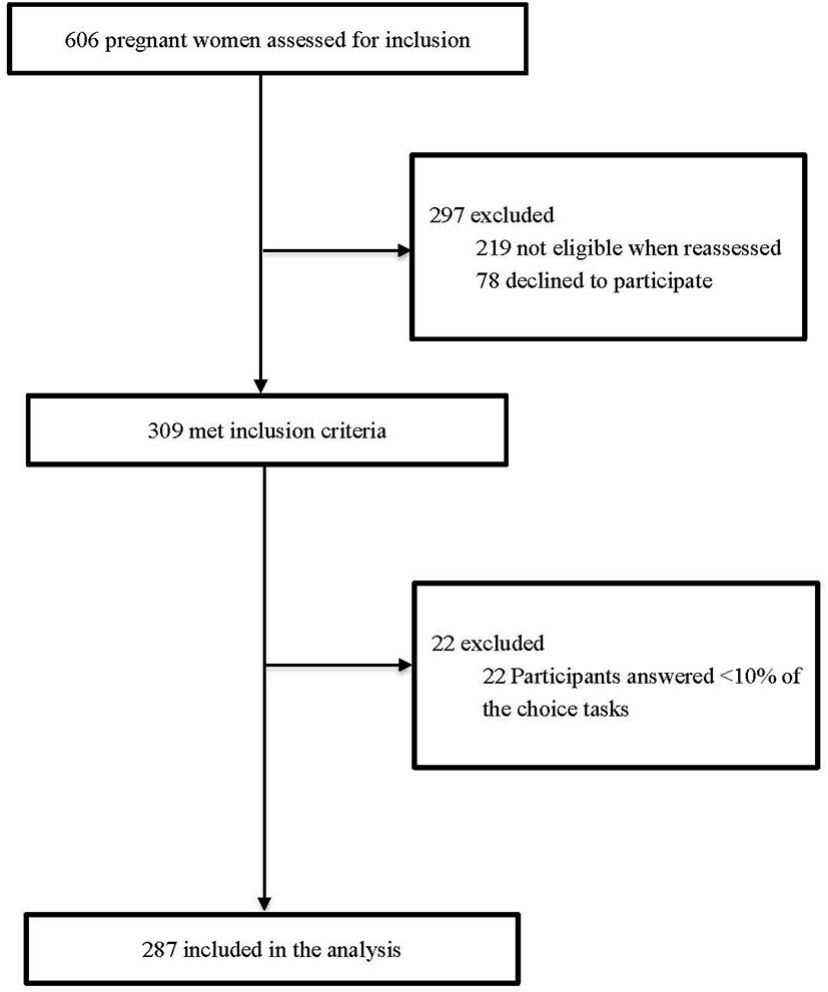
Flow chart of the subject selection.

A total of 309 pregnant women were initially recruited, and 93% (*n* = 287) of them completed the DCE survey (Figure 1 and Supplement 5). The detailed socio-demographic characteristics of these respondents are presented in Table 2. The mean age at enrollment for the included participants was 29.6 ± 5.4 years and the mean gestational week was 24.8±1.7. Over two thirds (66.9%) of the participants lived in villages and towns. The percentage of women with high school degree or above was 46.0%, 78.4% had more than one delivery experience, and a majority (72.5%) reported to have a household income ≤ CNY 60,000.

**Table 2:** Socio-demographic characteristics of respondents (*N* = 287).

**Characteristic**	**Mean**	**SD**
Age, years	29.6	5.4
Week of gestation, weeks	24.8	1.7
Household income, CNY^#^	52,600	35,200
Traffic cost, Yuan	11.1	29.8
Traffic time, minutes	31.9	50.5
Loss of working day, days	0.7	0.9
	***N***	**%**
**Area of residence**		
Living in the county	95	33.1
Living in the villages and towns (outside the county)	192	66.9
**Parity**		
Primipara	62	21.6
Multipara (≥2 times of gestation)	225	78.4
**Education**		
Primary school degree	11	2.8
Middle school degree	107	37.3
High school degree	38	13.2
Technical secondary school degree	46	16.0
2 year's college degree	48	16.7
4 years' university degree or above	37	12.9
**Occupation**		
Professional worker	26	9.1
Civil servant	11	3.8
Blue-collar worker	32	11.2
Farmer	93	32.4
Service personnel	15	5.2
Business owner	26	9.1
Unemployed	84	29.3
**Household income**		
≤ 30,000 CNY^#^	85	29.6
30,000~60,000 CNY	123	42.9
60,000~100,000 CNY	69	24.0
>100,000 CNY	10	3.5
**Medical insurance**		
New rural cooperative medical scheme	198	69.0
Urban employment medical insurance	63	22.0
Urban resident medical insurance	8	2.8
Others	18	6.3
**Maternity insurance (No)**	259	90.2

^#^1 Chinese Yuan (CNY) = USD 0.145 on January 2020.

### Discrete choice experiment results

#### Panel-mixed logistic model

The results of the panel-mixed logistic model are shown in Table 3. Five predefined attributes were associated with pregnant women's preferences. The participants preferred screening profiles that yielded a higher diagnostic rate (for example: 17.5 vs. 8.0%, OR: 2.89; 95% Confidence interval [CI]: 2.10, 3.96), reduced out-of-pocket costs (CNY 90 vs. CNY 10, OR: 0.37; 95%CI: 0.27, 0.49), shorter screening waiting-time (2.5 vs. 0.5 h, OR: 0.62; 95%CI: 0.49, 0.80), fewer hospital visits (2 vs. 1, OR: 0.71; 95%CI: 0.59, 0.85), and fewer number of blood draws (4 vs. 1, OR: 0.54; 95%CI: 0.43,0.68). The estimation of attributes did not change when we adjusted them by women's characteristics (Supplement 6), and our sample size were also sufficient to test these difference of attributes in *post hoc* analysis (Supplement 7).

**Table 3:** Attribute estimates of the Panel-mixed logistic model with observations = 4018.

			**Preference estimates**	
**Attributes**	**Coefficients**	**OR**	***P***	**Relative importance**
	**(95% CI)**	**(95% CI)**		
**Diagnostic rate**				
8.0%	0.00 (reference)	1.00 (reference)		
10.5%	0.20 (−0.04, 0.44)	1.22 (0.96, 1.55)	0.10	1
17.5%	1.06 (0.74, 1.38)	2.89 (2.10, 3.96)	< 0.001	
**Out-of-pocket cost** ^#^				
10 CNY	0.00 (reference)	1.00 (reference)		
30 CNY	−0.68 (−0.99, −0.37)	0.51 (0.37, 0.69)	< 0.0	2
60 CNY	−0.82 (−1.08, −0.57)	0.44 (0.34, 0.57)	< 0.001	
90 CNY	−1.00 (−1.30, −0.70)	0.37 (0.27, 0.49)	< 0.001	
**The number of blood draws**				
1 draw	0.00 (reference)	1.00 (reference)		
3 draws	−0.69 (−1.09, −0.30)	0.50 (0.34, 0.74)	< 0.00	3
4 draws	−0.61 (−0.84, −0.38)	0.54 (0.43, 0.68)	< 0.001	
**Screening waiting-time**				
0.5 h	0.00 (reference)	1.00 (reference)		
2.0 h	−0.24 (−0.44, −0.03)	0.79 (0.64, 0.97)	0.02	4
2.5 h	−0.47 (−0.72, −0.22)	0.62 (0.49, 0.80)	< 0.001	
**The number of hospital visits**				
1 visit	0.00 (reference)	1.00 (reference)		
2 visits	−0.34 (−0.53, −0.16)	0.71 (0.59, 0.85)	< 0.001	5
**Constant**	−1.71 (−2.29, −1.12)	0.18 (0.10, 0.33)	< 0.001	

Respondents: 287; Observations: 4018 (287^*^7^*^2).

^#^1 Chinese Yuan (CNY) = USD 0.145 on January 2020.

Model: number of blood draws, screening waiting-time, out-of-pocket cost, number of hospital visits, diagnostic rate.

The magnitude of differences in coefficients between the most and least favorable levels of the included attributes showed that the diagnostic rate was most influential in determining pregnant women's GDM screening preferences, followed by out-of-pocket costs, the number of blood draws, screening waiting-time and the number of hospital visits (Table 3).

#### Maternal changes of uptake probabilities in attributes

The changes of uptake probabilities that reflect the effectiveness of attributes on women's choice were presented in Table 4. When adjusting diagnostic rate from 8.0 to 17.5% (with other attributes set at mean values), women's uptake probabilities for this screening scenario substantially increased by 48.5% (95%CI: 36.4%, 60.6%). While the out-of-pocket cost had negative effect on the uptake probabilities when the cost increased from 10 CNY to 90 CNY (−46.3%, 95%CI: −58.0%, −34.5%). Similarly, the separate estimation of out-of-pocket costs, the number of blood draws, screening waiting-time, or the number of hospital visits showed that changes of the uptake probability changed a lot accordingly, and the variation also revealed the attributes' rank.

**Table 4:** Change (%) of uptake probabilities in attributes of GDM screening with observations = 4018.

**Changes from baseline**	**Change in probability**	**95% CI**	***P***
**Diagnostic rate**			
10.50%	9.8%	(−2.0%, 21.6%)	0.103
17.50%	48.5%	(36.4%, 60.6%)	0.000
**Out-of-pocket cost** ^#^			
30 CNY	−32.7%	(−46.6%, −18.7%)	0.000
60 CNY	−39.0%	(−49.8%, −28.2%)	0.000
90 CNY	−46.3%	(−58.0%, −34.5%)	0.000
**The number of blood draws**			
3 times	−33.4%	(−51.1%, −15.7%)	0.000
4 times	−30.0%	(−40.2%, −19.2%)	0.000
**Screening waiting-time**			
2.0 h	−11.9%	(−22.0%, −1.7%)	0.023
2.5 h	−23.2%	(−35.0%, −11.4%)	0.000
**The number of hospital visits**			
2 visits	−17.1%	(−25.9%, −8.2%)	0.000

Respondents: 287; Observations: 4018 (287^*^7^*^2).

^#^1 Chinese Yuan (CNY) = USD 0.145 on January 2020.

The baseline level: diagnostic rate 8.0%, our-of-pocket 10 CNY, number of blood draws 1, screening waiting-time 0.5 h, number of hospital visits 1.

With the inclusion of the specific attributes of “one-step” and “two-step” strategies, the changes of women's uptake probability from the full “two-step” strategy to the “one-step” strategy was 71.3% (95%CI: 52.2 to 90.1%). Notably, the results of testing investigation were consistent with the result of DCE (Supplement 8). Finally, there was no significant changes between the uptake probabilities of the “one-step” strategy and the first step of “two-step” strategy (−31.0%, 95%CI: −70.2 to 8.1%).

#### Interaction effects

Table 5 shows the association between individual characteristics and the women' preferences. We found that women with higher education preferred a screening scenario with higher diagnostic rate (OR: 4.28; *P* < 0.001) and less blood draws (OR: 4.86; *P* < 0.001 for four times blood draws). A similar result was also observed for women who were primipara than those who were multipara (OR: 2.74; *P* < 0.001). But the other individual characteristics had no obvious association with women preference on attributes. Pregnant women living in villages and towns tended to prefer fewer hospital visits and lower out-of-pocket costs compared to those living in the county.

**Table 5:** Results of interaction estimates of individual characteristics and the participant's preferences with observations=4018.

Attributes *characteristics	**Preference estimates**	
	**OR (95% CI)**	***P***
**Hospital visits** *areas		
One hospital visit * Living in the county	1.00 (reference)	
Two hospital visits * Living in the villages and towns	0.76 (0.53, 1.09)	0.136
Out-of-pockets*Areas		
10 CNY^#^ of out-of-pocket * Living in the county	1.00 (reference)	
30 CNY of out-of-pocket * Living in the villages and towns	0.87 (0.52, 1.42)	0.578
60 CNY of out-of-pocket * Living in the villages and towns	0.73 (0.41, 1.31)	0.293
90 CNY of out-of-pocket * Living in the villages	0.68 (0.41, 1.10)	0.120
**Diagnostic rate** *Parity		
8.0% * Multipara (≥2 times of gestation)	1.00 (reference)	
10.5% * Primipara	1.39 (0.88, 2.18)	0.153
17.5% * Primipara	2.74 (1.67, 4.49)	0.000
**The number of blood draws** *Education		
One time * High school degree below	1.00 (reference)	
Three times * University degree and above	3.33 (1.78, 6.21)	0.000
Four times * University degree and above	4.86 (2.15, 10.9)	0.000
Diagnostic rate*Education		
8.0% * High school degree below	1.00 (reference)	
10.5% * University degree and above	2.37 (1.19, 4.78)	0.015
17.5% * University degree and above	4.28 (2.02, 9.09)	0.000
**Waiting time** *Occupation		
0.5 h * Non-professional worker	1.00 (reference)	
2.0 h * Professional worker	1.51 (0.83, 2.72)	0.168
2.5 h * Professional worker	0.66 (0.27, 1.61)	0.364
Out-of-pocket cost*Household income		
10 CNY * ≤ 3,000 CNY^#^	1.00 (reference)	
30 CNY *>100,000 CNY	0.41 (0.11, 1.59)	0.201
60 CNY *>100,000 CNY	0.38 (0.07, 1.98)	0.252
90 CNY *>100,000 CNY	0.41 (0.10, 1.61)	0.207

^#^1 Chinese Yuan (CNY) = USD 0.145 on January 2020.

Model: number of blood draws, screening waiting-time, out-of-pocket cost, number of hospital visits, diagnostic rate, areas, parity, education, occupation, household income, hospital visits ^*^areas, out-of-pockets^*^areas, diagnostic rate ^*^parity, the number of blood draws ^*^ education, diagnostic rate^*^ education, waiting time ^*^occupation, out-of-pocket cost^*^household income.

## Discussion and conclusion

### Discussion

This study is the first to explore pregnant women's preferences for GDM screening from a patient perspective in rural China. Of the two routinely conducted (“one-step” and “two-step”) screening strategies in China, the “one-step” strategy was the overall preferred choice for pregnant Chinese women, with the diagnostic rate being the most influential attribute for pregnant women's preferences, followed by out-of-pocket costs, the number of blood draws, screening waiting-time and the number of hospital visits.

Currently, multiple screening methods exist worldwide and this major health services gap regarding an agreed screening method can lead to issues regarding the diagnosis and management of GDM. Achieving an agreement on GDM screening methods has been a major maternal healthcare challenge worldwide, especially in rural China. Rural China is confronted with a healthcare crisis in which the screening rate fails to keep pace with the incidence rate of GDM (23). Therefore, a major healthcare priority for Chinese women should be to increase GDM screening. We established that pregnant Chinese women's preference and acceptance are important factors to achieve an increased rate of GDM screening and treatment (22, 23). This study of patients' preferences has provided crucial evidence for comparing various screening methods for both the Chinese and international healthcare community.

In 2011, Chinese experts and professional institutions collaborated to develop a new guideline for GDM (42). This guideline suggests that the “one-step” strategy should be adopted in the developed areas of China, whereas the “two-step” could continue to be implemented in underdeveloped areas, considering women's economic conditions and willingness to pay. Importantly, these guidelines are not in line with our study's novel findings. More specifically, we did not observe any association between household income or living areas and women's preferences regarding out-of-pocket costs. We established that household income had no influence on maternal choices, even for pregnant women with lower socio-economic status which was supported by our Supplementary Table 8. Our pilot interview highlighted that with economic development, the successful implementation of poverty-alleviation policies, and increased importance attached to pregnancy in rural China, the costs of routine check-ups during pregnancy may pose only a minor barrier to health care access (43). Furthermore, our findings suggest that pregnant women in rural China preferred a “one-step” strategy to an entire “two-step” strategy. This result is consistent with many clinical and epidemiological studies which established that the “one-step” strategy is more effective in reducing complications during pregnancy (16, 44). For example, the leading HOPA study indicated that there is no lower threshold beyond which hyperglycemia during pregnancy is unproblematic for the offspring (16), and the “one-step” strategy with higher diagnostic rate could therefore reduce missed diagnosis and concomitant maternal and newborn complications. However, if women just need to receive GDM screening with the first step of “two-step” strategy, we find that the superiority of women's preference for “one-step” strategy is not obvious. But we found a huge preference gap between women requiring to receive the first step of “two-step” strategy and the entire “two-step” strategy, which explained the high rejection rate of the full “two-step” strategy among pregnant women in rural China. our previous investigation showed that there was a concerning phenomenon that a big proportion of pregnant women with abnormal glucose value diagnosed by the first step of “two-step” strategy rejected to visit hospital again to complete the second step of “two-step” strategy in rural China. Our results implied that the promotion of “one-step” screening strategy with higher diagnostic rate may significantly enhance the uptake and compliance of GDM screening among rural Chinese women; except for those with low GDM risk, and hence, having low probabilities to continue receiving the entire “two-step” GDM screening.

Our findings regarding attributes indeed demonstrated that pregnant women preferred screening methods with a higher diagnostic rate, and other attributes including out of pocket costs, the number of blood draws, screening waiting-time and the number of hospital visits were also influential, nevertheless, not as important as the diagnostic rate. Despite women have been informed in advance that a higher diagnostic rate might lead to misdiagnosis (which may mean they are treated for a condition they do not really have, or they do not receive the proper treatment/advice regarding their true condition), they were more concerned about the adverse health consequences of missed diagnosis compared with misdiagnosis (for example, macrosomia and neonatal hypoglycemia) (16). There is a possibility that participant might not all catch the true meaning of diagnostic rate, as we could not explain all important details to them due to certain limitations surrounding the complexity of the topic. Therefore, women' screening choices might have not been fully informed (even when they were warned of the possible “misdiagnosis” or “miss diagnosis” consequences of the included screening options). However, we do not expect this limitation to materially alter our results on diagnostic rate.

We also observed that women with their first pregnancy paid more attention to diagnostic rate than those with multiple gestation. We did not find any previous studies on the association between parity and diagnostic rate preferences, but some studies have shown a negative association between parity and screening rates (i.e., higher parity is associated with lower screening rates) (45). Psychological research during pregnancy indicated that women were more cautious and careful during their first pregnancy (46, 47), and they tended to consult more frequently before receiving a new test. In contrast, multipara paid less attention to this aspect due to their previous experience of safe childbirth (48). We suggest that this psychological phenomenon regarding pregnant women and their previous experience regarding gestation could partly explain our findings. As China now allows each family to have two children and subsequently more middle-aged women experience a second pregnancy, the incidence of GDM is likely to substantially rise in China in the next 5–10 years (49). Multipara's attention to GDM screening is important and could be improved by providing more information and education on the adverse health consequences of GDM and the subsequent benefits of improved blood glucose control.

The number of blood draws was identified as another important attribute. Our findings showed that more blood draws suggested a lower probability of pregnant women wanting GDM screening. This influence was also reflected in women's preferences for one step with a smaller number of blood draws. Some previous studies have indicated that blood draws might give rise to anxiety among pregnant women as they feel worried about the potential adverse consequences of multiple blood draws on the health of their babies (46). This finding indicates that increased consultation (such as the targeted sharing of validated information) and/or psychological counseling for pregnant women may increase rates of GDM screening. Previous studies have also shown that more psychological counseling will significantly increase these women's compliance (47).

Our study has several strengths. First, it uses DCE to elicit preferences, which takes into account patients' desires and feelings that are often ignored. Second, to improve the comprehension of DCE and the precision of parameter estimates in this study, a face-to-face pilot study was conducted in advance, and an explanation on how to complete the choice tasks as well as an example choice task were provided to the respondents. Third, even though our results are based on data from a single county in rural China and may have generalizability concerns, we do not regard it as a significant issue as women's maternal preference in rural China are not expected to materially differ from others. A report of Women's willingness on antenatal care in rural areas revealed that there are many aspects that are universal and consistent in China (50). Our preference findings of Chinese rural women have important representative value for healthcare decision-making.

This study also has some limitations. First, the investigation of the effects of various attributes using a hypothetical choice setting can result in hypothetical bias, as some hypothetical screening profiles may not exist in real-life situations. Second, our study did not consider the treatment costs deriving from false diagnoses (misdiagnosis or missed diagnosis), but we understand that these costs should be considered into policy making in the future. Third, the questionnaire was administrated and instructed by trained nurses who were familiar with participants, which may have led to some degree of response bias. Forth, compared with some nationally designated poor counties, the selected county in our study has relatively high socioeconomic and health outcomes so the generalizability of cost preference findings to pregnant women from poorer parts of rural China may be questioned, underlining the importance of conducting further studies in diverse parts of China. Finally, the preferences of women living in urban areas and other areas (west, south, and middle areas) areas are still unknown and should be investigated to enable rural vs. urban comparisons. Larger confirmatory studies with rural population are also recommended using data from multiple rural locations to validate/extend our exploratory findings.

## Conclusion

Pregnant women's preferences for GDM screening were associated with several attributes, with the diagnostic rate identified as the most important when choosing a screening method from patients' perspective. Our findings suggest that compared with the entire “two-step” strategy, the “one-step” strategy (with a higher diagnostic rate, lower out-of-pocket costs, fewer number of blood draws, shorter screening wait-time and fewer hospital visits) is more suitable to the circumstances of rural Chinese pregnant women, particularly for those with high GDM risk, low socioeconomic backgrounds and living in remote locations (i.e., villages and towns). This exploratory study provided a new direction to counter the negative influence of inconsistent GDM screening methods in China. We suggest a larger confirmatory in diverse regions study to validate our exploratory findings.

## Practice implications

The results provide insight that can be used to instruct the implementation of GDM screening for clinical practice, explore barriers to the promotion of GDM screening rate, and tailor screening advice based on individual characteristics that meet women' needs, especially the need to improve GDM management among people with risk factors of gestation diabetes in China.

## Data availability statement

The raw data supporting the conclusions of this article will be made available by the authors, without undue reservation.

## Ethics statement

The studies involving human participants were reviewed and approved by the Ethics Committee of Peking Union Medical College Hospital, Chinese Academy of Medical Sciences (Approval Number: ZS-1119). The patients/participants provided their written informed consent to participate in this study.

## Author contributions

TX conceptualized and designed the study, conducted the data analyses, wrote the first draft of the manuscript, and contributed to the interpretation of results. HF and AP reviewed and substantially revised the manuscript. QX, JC, and HA reviewed and edited the manuscript substantially. YJ, XG, DY, and LM contributed to the interpretation of results and critically reviewed and edited the manuscript for important intellectual content. XL contributed to the revision and improvement of the manuscript. All authors approved the final manuscript as submitted and agreed to be accountable for all aspects of the work.

## Funding

This study was funded by National Natural Science Foundation of China (NSFC Grant No. 71774006) and China Medical Board (CMB Grant No. 17-266) for HF; and by National Natural Science Foundation of China (NSFC Grant No. 72204172) for TX.

## Conflict of interest

The authors declare that the research was conducted in the absence of any commercial or financial relationships that could be construed as a potential conflict of interest.

## Publisher's note

All claims expressed in this article are solely those of the authors and do not necessarily represent those of their affiliated organizations, or those of the publisher, the editors and the reviewers. Any product that may be evaluated in this article, or claim that may be made by its manufacturer, is not guaranteed or endorsed by the publisher.

## Supplementary material

Supplement 1 A list of screening strategies produced by factorial designs.

Supplement 2 An example of a choice tasks.

Supplement 3 Detailed description of the statistical methods.

Supplement 4 Individual characteristic in interaction terms.

Supplement 5 Socio-demographic characteristics of 287 included respondents and 22 excluded participants.

Supplement 6 Attribute estimates of the Panel-mixed logistic model adjusted by women's characteristics.

Supplement 7 *Post-hoc* analysis for DCE sample.

Supplement 8 Results of interaction estimates of household income with five attributes.

Supplement 9 Maternal preferred choice for “one-step” and “two-step” strategy.

Supplement 10 DCE Questionnaire.
