# Supplementary material for: Maternal choices and preferences for screening strategies of gestational diabetes mellitus: A exploratory study using discrete choice experiment
Source: Front Public Health. 2022 Nov 1;10:864482. doi: 10.3389/fpubh.2022.864482 (PMC9664193; doi:10.3389/fpubh.2022.864482)
Supplement: Supplement 1 — A list of screening strategies produced by factorial designs. [file Data_Sheet_1.docx]

| **Supplement 1.** A list of screening strategies produced by factorial designs | | | | | |
| --- | --- | --- | --- | --- | --- |
| Screening options | Number of blood draws (times) | Screening waiting-time (hours) | Out-of-pocket cost (CNY)^#^ | Number of hospital visits (times) | GDM diagnostic rate (%) |
| 1 | 3 | 2.0 | 30 | 1 | 10.5 |
| 2 | 4 | 0.5 | 30 | 2 | 8.0 |
| 3 | 3 | 2.5 | 10 | 1 | 8.0 |
| 4 | 4 | 2.5 | 60 | 1 | 17.5 |
| 5 | 4 | 0.5 | 10 | 2 | 10.5 |
| 6 | 1 | 2.0 | 10 | 2 | 17.5 |
| 7 | 1 | 2.0 | 60 | 2 | 8.0 |
| 8 | 1 | 0.5 | 90 | 2 | 17.5 |
| 9 | 3 | 0.5 | 90 | 2 | 17.5 |
| 10 | 1 | 2.5 | 90 | 2 | 10.5 |
| 11 | 1 | 0.5 | 90 | 1 | 8.0 |
| 12 | 3 | 0.5 | 60 | 2 | 8.0 |
| 13 | 1 | 0.5 | 60 | 1 | 10.5 |
| 14 | 1 | 2.5 | 30 | 2 | 8.0 |
| 15 | 4 | 2.0 | 90 | 1 | 8.0 |
| 16 | 1 | 0.5 | 30 | 1 | 10.5 |
| ^#^1 Chinese Yuan (CNY) = US$ 0.145 on January 2020. | | | | | |

| **Supplement 2.** An example of a choice tasks | | |
| --- | --- | --- |
| **Attributes** | **Screening option 1** | **Screening option 2** |
| Number of blood draws | 3 times | 1 time |
| Screening waiting-time | 2.0 hours | 2.5 hours |
| Out-of-pocket cost^#^ | 30 CNY | 60 CNY |
| Number of hospital visits | 1 time | 2 times |
| GDM diagnostic rate | 10.5% | 17.5% |
|  |  |  |
| Which screening strategy do you prefer? |  |  |
| ^#^1 Chinese Yuan (CNY) = USD 0.145 on January 2020. | | |

**Supplement 3** Detailed description of the statistical methods

Demographic and attitudinal data was analyzed using descriptive statistics. Participants’ characteristics were presented as counts (N) and percentages (%) for categorical variables, and continuous data were described as mean and standard deviation (SD). Statistical analyses were performed in STATA version 17 (Stata Corp LP, College Station, TX, USA).

Discrete choice data was analyzed using the panel mixed logit (PML) models which accommodated the nature of the data [1]. STATA version 14 was used to construct the panel mixed logistic (PML) models that were estimated within this study. When using PML models, the data are adjusted for the panel structure (i.e., multilevel structure, the following Table). As every respondent completed 7 choice tasks, and that included 14 answers (also be explained 14 samples), these answers (samples) may be correlated. Instead of conditional logistic model assuming respondents have the same preferences and therefore makes the assumption of independence of irrelevant alternatives which may not be true, the PML model extends the standard conditional logistic model by allowing one or more of the parameters in the model to be randomly distributed and the coefficients in the model to vary across respondents. Moreover, PML models accounts for preference heterogeneity between respondents, i.e. respondents are allowed to have different preferences. And also, the PML models adjust the standard errors of utility estimates to account for repeated choices by the same individual. The model is estimated using the maximum simulated likelihood approach.

| **Table**. Data format | | | | |
| --- | --- | --- | --- | --- |
| ID | Question | Quality | Age | Pregnant week |
| 1 | 1 | 1 | 37 | 27 |
| 1 | 2 | 1 | 37 | 27 |
| 1 | 3 | 1 | 37 | 27 |
| 1 | 4 | 1 | 37 | 27 |
| 1 | 5 | 1 | 37 | 27 |
| 1 | 6 | 1 | 37 | 27 |
| 1 | 7 | 1 | 37 | 27 |
| 1 | 8 | 1 | 37 | 27 |
| 1 | 9 | 1 | 37 | 27 |
| 1 | 10 | 1 | 37 | 27 |
| 1 | 11 | 1 | 37 | 27 |
| 1 | 12 | 1 | 37 | 27 |
| 1 | 13 | 1 | 37 | 27 |
| 1 | 14 | 1 | 37 | 27 |
| 2 | 1 | 1 | 28 | 26 |
| 2 | 2 | 1 | 28 | 26 |
| 2 | 3 | 1 | 28 | 26 |
| 2 | 4 | 1 | 28 | 26 |
| 2 | 5 | 1 | 28 | 26 |
| 2 | 6 | 1 | 28 | 26 |
| 2 | 7 | 1 | 28 | 26 |
| 2 | 8 | 1 | 28 | 26 |
| 2 | 9 | 1 | 28 | 26 |
| 2 | 10 | 1 | 28 | 26 |
| 2 | 11 | 1 | 28 | 26 |
| 2 | 12 | 1 | 28 | 26 |
| 2 | 13 | 1 | 28 | 26 |
| 2 | 14 | 1 | 28 | 26 |

In the analyses, all attributes were specified as random coefficients, and choice scenarios were identified using a grouping variable. Then a higher level grouping was specified at the level of the respondent to account for multiple choice scenarios per respondent and to account for preference heterogeneity [2].

The theoretical model describing the utility of screening profiles was based on the attributes as follows:

U = $\hat{\beta}$0 +$\hat{\beta}$1 *(3 blood draws) + $\hat{\beta}$2 *(4 blood draws) + $\hat{\beta}$3 *(CNY 30 out-of-pocket costs) + $\hat{\beta}$4 *(CNY 60 out-of-pocket costs) + $\hat{\beta}$5 *(CNY 90 out-of-pocket costs) + $\hat{\beta}$6 *(2 hours screening waiting-time) + $\hat{\beta}$7 *(2.5 hours screening waiting-time) + $\hat{\beta}$8 *(2 hospital visits) + $\hat{\beta}$9 *(10.5% diagnostic rate) + $\hat{\beta}$10 *(17.5% diagnostic rate) + $\hat{\beta}$11*attributes*individual characteristics + ε.

U describes the utility of a specific screening profile based on the attributes that were included in the DCE. The dependent variable represented whether a particular screening profile was chosen. The independent variables were the attribute levels that made up the screening profile [3]. β ̂0 represented the alternative specific constant, β ̂1 to β ̂10 were the attribute estimates that indicated the relative importance of each attribute. A positive regression coefficient (β ̂) indicated that pregnant women preferred that level within an attribute, whereas a negative coefficient suggested that they did not prefer that level within an attribute.

Difference in coefficients (as preference weights) between the most and least favorable levels of an attribute was interpreted as the relative importance of this attribute.

We also assumed that individual characteristics, such as living areas, parity, education level and household income, yielded differing influences on pregnant women’s preferences (Supplement 4). β ̂11 was the estimate for the interaction between attributes and the individual characteristics.

We further estimated the marginal uptake probabilities when one of attributes changed from low level to higher and other attributes were default (at mean values) or at specified values. The method and mechanism of marginal uptake probabilities calculation followed WHO DCE guidelines. The formula is based on regression coefficient (β ̂) of DCE:

$$P_{i}=\frac{e^{\beta^{'}x_{i}}}{\sumⅇ^{\beta^{'}x_{j}}}$$

Where Pi was the changes of uptake probability from a screening profile j to another screening profile i. We achieved this process by using code of “nlcom(exp(_b[x_11_])-exp(_b[x_12_]))/(exp(_b[x_11_])+exp(_b[x_12_])) (x_11_=level 1; x_12_=level2…. )” in Stata 17.0. For example, we calculated the changes of probabilities when diagnosed rate increase to 17.5% from 8%. We use the code of “nlcom(exp(_b[3.srate])-exp(_b[1.srate]))/(exp(_b[3.srate])+exp(_b[1.srate]))”.

The changes of uptake probability of pregnant women from the least favorable attribute (8% diagnostic rate, CNY 90 out-of-pocket costs, four blood draw, two hospital visit, and 2.5 hour screening waiting-time) to the most favorable attributes (17.5% diagnostic rate, CNY 10 out-of-pocket costs, one blood draw, one hospital visit, and 0.5 hour screening waiting-time) were separately estimated.

We also estimate the changes of women’s uptake probabilities for the “one-step” strategy (with attributes: 17.5% diagnostic rate, CNY 30 out-of-pocket costs, three blood draws, one hospital visit, and two hours screening waiting-time) from first step of “two-step” strategy (with attributes: 8% diagnostic rate, CNY 10 out-of-pocket costs, one blood draw, one hospital visit, and 0.5 hour screening waiting-time); and from the entire “two-step” strategy (with 8% diagnostic rate, CNY 60 out-of-pocket costs, four blood draws, two hospital visits, and 2.5 hours screening waiting-time).

**Reference**

1. Revelt D, Train KE. Mixed Logit with Repeated Choices: Households' Choices of Appliance Efficiency Level. The Review of Economics and Statistics 1998; 80: 647-57.

2. Ryan M, Gerard K, Amaya-Amaya M: Using Discrete Choice Experiments to Value Health and Health Care. Dordrecht: Springer; 2008.

3. Bech M, Gyrd-Hansen D. Effects coding in discrete choice experiments. Health Econ 2005;14: 1079-83.

| **Supplement 4.** Individual characteristic in interaction terms | | |
| --- | --- | --- |
| **Individual characteristic** | **Level** | **Value** |
| Living areas | 1 | Living in the villages and towns |
|  | 0 | Living in the county |
| Parity | 1 | Multipara (≥2 times of gestation) |
|  | 0 | Primipara |
| Education level | 1 | High school degree or below |
|  | 2 | Senior high school degree or university associate degree |
|  | 3 | University bachelor’s degree or above |
| Occupation | 1 | Professional worker |
|  | 0 | Others |
| Household income^#^ | 1 | 0-30000 CNY |
|  | 2 | 30000-60000 CNY |
|  | 3 | 60000-100000 CNY |
|  | 4 | >100000 CNY |
| ^#^1 Chinese Yuan (CNY) = USD 0.145 on January 2020. | | |

| **Supplement 5.** Socio-demographic characteristics of 287 included respondents and 22 excluded participants. | | | |
| --- | --- | --- | --- |
|  | **Included** | **Excluded** |  |
| **Characteristic** | **Mean (SD)** | **Mean (SD)** | **P value/χ^2^** |
| **Age, years** | 29.6 (5.4) | 30.1 (3.5) | 0.66 |
| **Week of gestation, weeks** | 24.8 (1.7) | 25.5 (1.3) | 0.04 |
| **Household income, CNY**^#^ | 52,600 (35,200) | 62,350 (43,503) | 0.20 |
| **Traffic cost, Yuan** | 11.1 (29.8) | 13.1(28.8) | 0.75 |
| **Traffic time, minutes** | 31.9 (50.5) | 32.4 (27.1) | 0.96 |
| **Loss of working day, days** | 0.7 (0.9) | 0.6 (0.6) | 0.37 |
|  | **N (%)** | **N (%)** |  |
| **Areas** |  |  |  |
| Living in the county | 95 (33.1) | 9 (40.9) | 0.45 |
| Living in the villages and towns (outside the county) | 192 (66.9) | 13 (59.1) |  |
| **Parity** |  |  |  |
| Primipara | 62 (21.6) | 1 (4.5) | 0.32 |
| Multipara (≥2 times of gestation) | 225 (78.4) | 21 (95.5) |  |
| **Education** |  |  |  |
| High school degree and below | 156 (54.4) | 13 (59.1) | 0.51 |
| Senior high school degree or university associate degree | 94 (32.8) | 8 (36.4) |  |
| University bachelor’s degree or above | 37 (12.9) | 1 (4.5) |  |
| **Occupation** |  |  |  |
| Professional worker | 26 (9.1) | 2 (9.1) | 0.99 |
| Others | 261 (91.9) | 20 (91.9) |  |
| **Household income** |  |  |  |
| ≤30,000 CNY* | 85 (29.6) | 5 (22.7) | 0.53 |
| 30,000~60,000 CNY | 123 (42.9) | 8 (36.4) |  |
| 60,000~100,000 CNY | 69 (24.0) | 5 (22.7) |  |
| >100,000 CNY | 10 (3.5) | 2 (9.1) |  |
| ^#^1 Chinese Yuan (CNY) = USD 0.145 on January 2020. | | | |

| **Supplement 6.** Attribute estimates of the Panel-mixed logistic model adjusted by women’s characteristics | | | | | |
| --- | --- | --- | --- | --- | --- |
|  |  | | **Preference estimates** | | |
| **Attributes** | Coefficients (95% CI) | OR (95% CI) | | P-value | Relative Importance |
| **Diagnostic rate** |  |  | |  |  |
| 8.0% | 0.00 (reference) | 1.00 (reference) | |  |  |
| 10.5% | 0.20 (-0.04, 0.44) | 1.22 (0.96, 1.55) | | 0.105 | 1 |
| 17.5% | 1.06 (0.74, 1.38) | 2.89 (2.10, 3.96) | | 0.000 |  |
| **Out-of-pocket cost**^#^ |  |  | |  |  |
| 10 CNY | 0.00 (reference) | 1.00 (reference) | |  |  |
| 30 CNY | -0.68 (-0.99, -0.37) | 0.51 (0.37, 0.69) | | 0.000 | 2 |
| 60 CNY | -0.82 (-1.08, -0.57) | 0.44 (0.34, 0.57) | | 0.000 |  |
| 90 CNY | -1.00 (-1.30, -0.70) | 0.37 (0.27, 0.49) | | 0.000 |  |
| **The number of blood draws** |  |  | |  |  |
| 1 | 0.00 (reference) | 1.00 (reference) | |  |  |
| 3 | -0.69 (-1.09, -0.30) | 0.50 (0.34, 0.74) | | 0.000 | 3 |
| 4 | -0.61 (-0.84, -0.38) | 0.54 (0.43, 0.68) | | 0.000 |  |
| **Screening waiting-time** |  |  | |  |  |
| 0.5 hour | 0.00 (reference) | 1.00 (reference) | |  |  |
| 2.0 hours | -0.24 (-0.44, -0.03) | 0.79 (0.64, 0.97) | | 0.024 | 4 |
| 2.5 hours | -0.47 (-0.72, -0.22) | 0.62 (0.49, 0.80) | | 0.000 |  |
| **The number of hospital visits** |  |  | |  |  |
| 1 | 0.00 (reference) | 1.00 (reference) | |  |  |
| 2 | -0.34 (-0.53, -0.16) | 0.71 (0.59, 0.85) | | 0.000 | 5 |
| **Constant** | -1.71 (-2.29, -1.12) | 0.18 (0.10, 0.33) | | 0.000 |  |
| Note: Respondents: 287; Observations: 4018 (287*7*2)  ^#^1 Chinese Yuan (CNY) = USD 0.145 on January 2020.  Model adjusted by living areas, parity, educational level, occupation, household income. | | | | | |

**Supplement 7** Post hoc analysis for DCE sample

| Power | Number | X=1 | P0 | P1 | OR | Alpha | Beta |
| --- | --- | --- | --- | --- | --- | --- | --- |
| 0.8006 | 186 | 10 | 0.5 | 0.091 | 0.1 | 0.05 | 0.1994 |
| 0.8022 | 80 | 25 | 0.5 | 0.091 | 0.1 | 0.05 | 0.1978 |
| 0.8027 | 48 | 50 | 0.5 | 0.091 | 0.1 | 0.05 | 0.1973 |
| 0.8509 | 213 | 10 | 0.5 | 0.091 | 0.1 | 0.05 | 0.1491 |
| 0.85 | 91 | 25 | 0.5 | 0.091 | 0.1 | 0.05 | 0.15 |
| 0.853 | 55 | 50 | 0.5 | 0.091 | 0.1 | 0.05 | 0.147 |
| 0.9004 | 249 | 10 | 0.5 | 0.091 | 0.1 | 0.05 | 0.0996 |
| 0.9014 | 107 | 25 | 0.5 | 0.091 | 0.1 | 0.05 | 0.0986 |
| 0.9008 | 64 | 50 | 0.5 | 0.091 | 0.1 | 0.05 | 0.0992 |
| 0.8001 | 808 | 10 | 0.5 | 0.333 | 0.5 | 0.05 | 0.1999 |
| 0.8008 | 382 | 25 | 0.5 | 0.333 | 0.5 | 0.05 | 0.1992 |
| 0.8004 | 278 | 50 | 0.5 | 0.333 | 0.5 | 0.05 | 0.1996 |
| 0.85 | 924 | 10 | 0.5 | 0.333 | 0.5 | 0.05 | 0.15 |
| 0.8508 | 437 | 25 | 0.5 | 0.333 | 0.5 | 0.05 | 0.1492 |
| 0.8503 | 318 | 50 | 0.5 | 0.333 | 0.5 | 0.05 | 0.1497 |
| 0.9002 | 1082 | 10 | 0.5 | 0.333 | 0.5 | 0.05 | 0.0998 |
| 0.9004 | 511 | 25 | 0.5 | 0.333 | 0.5 | 0.05 | 0.0996 |
| 0.9002 | 372 | 50 | 0.5 | 0.333 | 0.5 | 0.05 | 0.0998 |
| 0.8001 | 2202 | 10 | 0.5 | 0.6 | 1.5 | 0.05 | 0.1999 |
| 0.8003 | 1051 | 25 | 0.5 | 0.6 | 1.5 | 0.05 | 0.1997 |
| 0.8001 | 780 | 50 | 0.5 | 0.6 | 1.5 | 0.05 | 0.1999 |
| 0.8501 | 2519 | 10 | 0.5 | 0.6 | 1.5 | 0.05 | 0.1499 |
| 0.8502 | 1202 | 25 | 0.5 | 0.6 | 1.5 | 0.05 | 0.1498 |
| 0.8504 | 893 | 50 | 0.5 | 0.6 | 1.5 | 0.05 | 0.1496 |
| 0.9001 | 2948 | 10 | 0.5 | 0.6 | 1.5 | 0.05 | 0.0999 |
| 0.9002 | 1407 | 25 | 0.5 | 0.6 | 1.5 | 0.05 | 0.0998 |
| 0.9 | 1044 | 50 | 0.5 | 0.6 | 1.5 | 0.05 | 0.1 |
| 0.8001 | 808 | 10 | 0.5 | 0.667 | 2 | 0.05 | 0.1999 |
| 0.8008 | 382 | 25 | 0.5 | 0.667 | 2 | 0.05 | 0.1992 |
| 0.8004 | 278 | 50 | 0.5 | 0.667 | 2 | 0.05 | 0.1996 |
| 0.85 | 924 | 10 | 0.5 | 0.667 | 2 | 0.05 | 0.15 |
| 0.8508 | 437 | 25 | 0.5 | 0.667 | 2 | 0.05 | 0.1492 |
| 0.8503 | 318 | 50 | 0.5 | 0.667 | 2 | 0.05 | 0.1497 |
| 0.9002 | 1082 | 10 | 0.5 | 0.667 | 2 | 0.05 | 0.0998 |
| 0.9004 | 511 | 25 | 0.5 | 0.667 | 2 | 0.05 | 0.0996 |
| 0.9002 | 372 | 50 | 0.5 | 0.667 | 2 | 0.05 | 0.0998 |
| 0.8003 | 376 | 10 | 0.5 | 0.75 | 3 | 0.05 | 0.1997 |
| 0.8013 | 174 | 25 | 0.5 | 0.75 | 3 | 0.05 | 0.1987 |
| 0.802 | 122 | 50 | 0.5 | 0.75 | 3 | 0.05 | 0.198 |
| 0.8502 | 430 | 10 | 0.5 | 0.75 | 3 | 0.05 | 0.1498 |
| 0.8511 | 199 | 25 | 0.5 | 0.75 | 3 | 0.05 | 0.1489 |
| 0.8504 | 139 | 50 | 0.5 | 0.75 | 3 | 0.05 | 0.1496 |
| 0.9006 | 504 | 10 | 0.5 | 0.75 | 3 | 0.05 | 0.0994 |
| 0.901 | 233 | 25 | 0.5 | 0.75 | 3 | 0.05 | 0.099 |
| 0.9009 | 163 | 50 | 0.5 | 0.75 | 3 | 0.05 | 0.0991 |
| 0.8008 | 274 | 10 | 0.5 | 0.8 | 4 | 0.05 | 0.1992 |
| 0.8004 | 124 | 25 | 0.5 | 0.8 | 4 | 0.05 | 0.1996 |
| 0.8013 | 84 | 50 | 0.5 | 0.8 | 4 | 0.05 | 0.1987 |
| 0.8503 | 313 | 10 | 0.5 | 0.8 | 4 | 0.05 | 0.1497 |
| 0.8507 | 142 | 25 | 0.5 | 0.8 | 4 | 0.05 | 0.1493 |

| **Supplement 8.** Results of interaction estimates of household income with five attributes | | |
| --- | --- | --- |
|  | **Preference estimates** | |
| **Attributes * characteristics** | **OR (95% CI)** | **P-value** |
| **Hospital visits *** **Household income** |  |  |
| One hospital visit * ≤30,000 CNY# | 1.00 (reference) |  |
| Two hospital visits* 30,000-60,000 CNY | 0.81 (0.52,1.25) | 0.34 |
| Two hospital visits*60,000-100,000 CNY | 1.22 (0.75,2.00) | 0.41 |
| Two hospital visits*>100,000 CNY | 1.37 (0.44, 4.32) | 0.59 |
| **Diagnostic rate *** **Household income** |  |  |
| 8.0%* ≤30,000 CNY | 1.00 (reference) |  |
| 10.5%* 30,000-60,000 CNY | 1.09 (0.66,1.80) | 0.71 |
| 10.5% * 60,000-100,000 CNY | 0.96 (0.55,1.67) | 0.90 |
| 10.5% * >100,000 CNY | 1.13 (0.31, 4.09) | 0.84 |
| 17.5%* 30,000-60,000 CNY | 1.28 (0.72, 2.25) | 0.39 |
| 17.5% * 60,000-100,000 CNY | 0.88 (0.46, 1.67) | 0.70 |
| 17.5% * >100,000 CNY | 3.42 (0.47,24.5) | 0.22 |
| **The number of blood draws *Household income** |  |  |
| One time * ≤30,000 CNY | 1.00 (reference) |  |
| Three times *30,000-60,000 CNY | 1.51 (0.95,2.40) | 0.07 |
| Three times * 60,000-100,000 CNY | 1.48 (0.88,2.49) | 0.13 |
| Three times * >100,000 CNY | 0.72 (0.15,3.33) | 0.67 |
| Four times *30,000-60,000 CNY | 1.50 (0.85, 2.63) | 0.15 |
| Four times * 60,000-100,000 CNY | 2.06 (0.89, 3.85) | 0.12 |
| Four times * >100,000 CNY | 0.70 (0.13,3.74) | 0.68 |
| **Waiting time *** **Household income** |  |  |
| 0.5 hour * ≤30,000 CNY | 1.00 (reference) |  |
| 2.0 hours * 30,000-60,000 CNY | 0.94 (0.58,1.50) | 0.79 |
| 2.0 hours* 60,000-100,000 CNY | 0.92 (0.54,1.57) | 0.77 |
| 2.0 hours* >100,000 CNY | 1.93 (0.42,8.88) | 0.39 |
| 2.5 hours * 30,000-60,000 CNY | 0.70 (0.40, 1.21) | 0.21 |
| 2.5 hours* 60,000-100,000 CNY | 0.91 (0.50, 1.66) | 0.77 |
| 2.5 hours* >100,000 CNY | 0.67 (0.16,2.66) | 0.57 |
| **Out-of-pocket cost*Household income** |  |  |
| 10 CNY *≤30,000 CNY^#^ | 1.00 (reference) |  |
| 30 CNY * 30,000-60,000 CNY | 0.98 (0.53,2.33) | 0.95 |
| 30 CNY*60,000-100,000 CNY | 1.17 (0.59,1.98) | 0.64 |
| 30 CNY *>100,000 CNY | 0.30 (0.05,1.86) | 0.20 |
| 60 CNY * 30,000-60,000 CNY | 1.42 (0.76, 2.63) | 0.26 |
| 60 CNY*60,000-100,000 CNY | 1.54 (0.77,3.11) | 0.22 |
| 60 CNY *>100,000 CNY | 0.25 (0.03, 1.84) | 0.18 |
| 90 CNY * 30,000-60,000 CNY | 0.94 (0.52,1.69) | 0.84 |
| 90 CNY*60,000-100,000 CNY | 1.08 (0.56, 2.09) | 0.82 |
| 90 CNY *>100,000 CNY | 0.25 (0.04, 1.65) | 0.15 |
| ^#^1 Chinese Yuan (CNY) = USD 0.145 on January 2020.  Model: number of blood draws, screening waiting-time, out-of-pocket cost, number of hospital visits, diagnostic rate, areas, parity, education, occupation, household income, hospital visits *areas, out-of-pockets*areas, diagnostic rate *parity, the number of blood draws * education, diagnostic rate* education, waiting time *occupation, out-of-pocket cost*household income | | |

| **Supplement 9.** Maternal preferred choice for “one-step” and “two-step” strategy | | | |
| --- | --- | --- | --- |
| **Strategy** | **N** | **%** | **P-value** |
| “one-step” strategy | 215 | 74.91% | **<0.001** |
| “two-step” strategy | 70 | 24.39% |  |
| Missing data | 2 | 0.70% |  |

# Supplement 10. Questionnaires

Date：_____ Year ____ Month ____Day

**Questionnaire**

**Maternal Characteristic, Preferences and Willingness to Pay for Screening Strategies of Gestational Diabetes Mellitus in Rural China**

Questionnaire Number: __________

Investigator Name: _______________

Screening of Pregnant Women（1.One-step 2.Part Two-step 3. Entire Two-step）

Respondent Name: __________

Respondent Phone Number：_____________ ID ----------------------------------

**Quality of Performance：1 Good 2 Medium 3 Bad**

**Group Leader Signature: _________**

1. **Characteristics**

|  | **Survey Content** | **Answer** |
| --- | --- | --- |
| 1 | Pregnant women’s age： |  |
| 2 | Pregnancy week： |  |
| 3 | Parity： |  |
| 4 | Living areas：1. County 2. Rural |  |
| 5 | Education：1. Illiteracy 2. Primary School 3. Middle School 4. High School 5. Technical Secondary School 6. College 7. Undergraduate 8. Graduate And Above |  |
| 6 | Occupation：1. Professional technician (Doctor, Professor, Lawyer, Architect, Engineer) 2. Civil servant 3. Worker 4. Farmer 5. Service Worker 6. Self-employed or private 7. Student 8. Other |  |
| 7 | Mean work day of a week ________day，mean work time of a Day _______h |  |
| 8 | Your annual income last year： |  |
| 9 | Total annual household income last year： |  |
| 10 | The number of people living in your family： |  |
| 11 | Medical insurance: 1. No 2. New Rural Cooperative Medical Care 3. Urban Employee Medical Insurance 4. Urban Residents Medical Insurance 5. Commercial Medical Insurance 6. Public Medical Insurance 7. Others |  |
| 12 | Do you have fertility insurance? |  |
| 13 | Transportation costs for this screening ________Yuan |  |
| 14 | How long will it delay you work time for this screening ________day |  |

1. **Pregnant Women’s Cognition of Gestational Diabetes**

|  | **Survey Content** | **Answer** |
| --- | --- | --- |
| 1 | Do you know about gestational diabetes?  1. Knowing clearly 2. Knowing relatively clear 3. Generally 4. Not very clear (jump to question 3) 5. Not at all clear (jump to question 3) |  |
| 2 | How did you find out this information about gestational diabetes?   1. By medical staff 2 By books, media 3. By listening to other pregnant women, friends or relatives, etc   4. Other _________ (multi-optional) |  |
| 3 | Do you know about the possible complications of gestational diabetes in pregnant women?  1. Knowing clearly 2. Knowing relatively clear 3. Generally 4. Not very clear (jump to question 5) 5. Not at all clear (jump to question 5) |  |
| 4 | How did you find out this information about complications?   1. By medical staff 2 By books, media 3. By listening to other pregnant women, friends or relatives, etc   4. Other _________ (multi-optional) |  |
| 5 | Do you know the purpose of this gestational diabetes screening?  1. Knowing clearly 2. Knowing relatively clear 3. Generally 4. Not very clear (jump to question 7) 5. Not at all clear (jump to question 7) |  |
| 6 | How did you find out this information about gestational diabetes screening?   1. By medical staff 2 By books, media 3. By listening to other pregnant women, friends or relatives, etc   4. Other _________ (multi-optional) |  |
| 7 | Do you think it is important of this screening for gestational diabetes?  1. Very important 2. Relatively important 3. Generally 4. Not very clear 5. Not at all clear |  |

**3. Measurement of maternal preference for screening method (discrete choice experiment A)**

1)

|  | **75gOGTT（one-step）** | **50gOGT（two-step）** |
| --- | --- | --- |
| Times of drawing blood | 3 | 4 |
| Waiting time for screening | 2h | 0.5h |
| Out-of-pocket cost | 30 | 30 |
| The number of hospital visits | 1 | 2 |
| GDM diagnosing rate | 10.5% | 8% |
| Which screening strategy do you prefer to choose？ |  |  |

2)

|  | **75gOGTT（one-step）** | **50gOGT（two-step）** |
| --- | --- | --- |
| Times of drawing blood | 3 | 3 |
| Waiting time for screening | 2h | 2.5h |
| Out-of-pocket cost | 30 | 10 |
| The number of hospital visits | 1 | 1 |
| GDM diagnosing rate | 10.5% | 8% |
| Which screening strategy do you prefer to choose？ |  |  |

3)

|  | **75gOGTT（one-step）** | **50gOGT（two-step）** |
| --- | --- | --- |
| Times of drawing blood | 3 | 4 |
| Waiting time for screening | 2h | 2.5 |
| Out-of-pocket cost | 30 | 60 |
| The number of hospital visits | 1 | 1 |
| GDM diagnosing rate | 10.5% | 17.5% |
| Which screening strategy do you prefer to choose？ |  |  |

4)

|  | **75gOGTT（one-step）** | **50gOGT（two-step）** |
| --- | --- | --- |
| Times of drawing blood | 3 | 4 |
| Waiting time for screening | 2h | 0.5h |
| Out-of-pocket cost | 30 | 10 |
| The number of hospital visits | 1 | 2 |
| GDM diagnosing rate | 10.5% | 10.5% |
| Which screening strategy do you prefer to choose？ |  |  |

5)

|  | **75gOGTT（one-step）** | **50gOGT（two-step）** |
| --- | --- | --- |
| Times of drawing blood | 3 | 1 |
| Waiting time for screening | 2h | 2h |
| Out-of-pocket cost | 30 | 10 |
| The number of hospital visits | 1 | 2 |
| GDM diagnosing rate | 10.5% | 17.5% |
| Which screening strategy do you prefer to choose？ |  |  |

6)

|  | **75gOGTT（one-step）** | **50gOGT（two-step）** |
| --- | --- | --- |
| Times of drawing blood | 3 | 1 |
| Waiting time for screening | 2h | 2h |
| Out-of-pocket cost | 30 | 60 |
| The number of hospital visits | 1 | 2 |
| GDM diagnosing rate | 10.5% | 8% |
| Which screening strategy do you prefer to choose？ |  |  |

7)

|  | **75gOGTT（one-step）** | **50gOGT（two-step）** |
| --- | --- | --- |
| Times of drawing blood | 3 | 1 |
| Waiting time for screening | 2h | 0.5h |
| Out-of-pocket cost | 30 | 10 |
| The number of hospital visits | 1 | 1 |
| GDM diagnosing rate | 10.5% | 8% |
| Which screening strategy do you prefer to choose？ |  |  |

1. **Measurement of maternal preference for screening method (discrete choice experiment B)**

1)

|  | **75gOGTT（one-step）** | **50gOGT（two-step）** |
| --- | --- | --- |
| Times of drawing blood | 3 | 1 |
| Waiting time for screening | 0.5h | 2.5h |
| Out-of-pocket cost | 90 | 90 |
| The number of hospital visits | 2 | 2 |
| GDM diagnosing rate | 17.5% | 10.5% |
| Which screening strategy do you prefer to choose？ |  |  |

2)

|  | **75gOGTT（one-step）** | **50gOGT（two-step）** |
| --- | --- | --- |
| Times of drawing blood | 3 | 1 |
| Waiting time for screening | 0.5h | 0.5h |
| Out-of-pocket cost | 90 | 90 |
| The number of hospital visits | 2 | 1 |
| GDM diagnosing rate | 17.5% | 8% |
| Which screening strategy do you prefer to choose？ |  |  |

3)

|  | **75gOGTT（one-step）** | **50gOGT（two-step）** |
| --- | --- | --- |
| Times of drawing blood | 3 | 3 |
| Waiting time for screening | 0.5h | 0.5h |
| Out-of-pocket cost | 90 | 60 |
| The number of hospital visits | 2 | 2 |
| GDM diagnosing rate | 17.5% | 8% |
| Which screening strategy do you prefer to choose？ |  |  |

4)

|  | **75gOGTT（one-step）** | **50gOGT（two-step）** |
| --- | --- | --- |
| Times of drawing blood | 3 | 1 |
| Waiting time for screening | 0.5h | 0.5h |
| Out-of-pocket cost | 90 | 60 |
| The number of hospital visits | 2 | 1 |
| GDM diagnosing rate | 17.5% | 10.5% |
| Which screening strategy do you prefer to choose？ |  |  |

5)

|  | **75gOGTT（one-step）** | **50gOGT（two-step）** |
| --- | --- | --- |
| Times of drawing blood | 3 | 1 |
| Waiting time for screening | 0.5h | 2.5h |
| Out-of-pocket cost | 90 | 30 |
| The number of hospital visits | 2 | 2 |
| GDM diagnosing rate | 17.5% | 8% |
| Which screening strategy do you prefer to choose？ |  |  |

6)

|  | **75gOGTT（one-step）** | **50gOGT（two-step）** |
| --- | --- | --- |
| Times of drawing blood | 3 | 4 |
| Waiting time for screening | 0.5h | 2h |
| Out-of-pocket cost | 90 | 90 |
| The number of hospital visits | 2 | 1 |
| GDM diagnosing rate | 17.5% | 8% |
| Which screening strategy do you prefer to choose？ |  |  |

7)

|  | **75gOGTT（one-step）** | **50gOGT（two-step）** |
| --- | --- | --- |
| Times of drawing blood | 3 | 1 |
| Waiting time for screening | 0.5h | 0.5h |
| Out-of-pocket cost | 90 | 30 |
| The number of hospital visits | 2 | 1 |
| GDM diagnosing rate | 17.5% | 10.5% |
| Which screening strategy do you prefer to choose？ |  |  |

1. **Willingness to pay**

| **Gestational Diabetes Mellitus (GDM)**: Gestational diabetes mellitus (GDM) is a common and risk pregnancy disease, which is closely related to the occurrence of adverse pregnancy outcomes such as macrosomia, premature delivery, and also increasing cesarean section rate and preeclampsia rate. A large number of clinical studies have shown that if there are no timely and scientific screening and intervention taken for patients with gestational diabetes, the morbidity and mortality of maternal and perinatal complications rate will increase. The fetus is prone to be macrosomia, and also have hyperbilirubinemia, neonatal distress, neonatal hypoglycemia, asphyxia, stillbirth, teratoma and other complications. Mothers are prone to occur preeclampsia, postpartum hemorrhage, premature rupture, infection, cesarean section, hypohydremia, abortion, ketosis and other complications, which will cause great pain and burden for the maternal family  **The Importance of Screening:** Interventions for the diagnosis and treatment of gestational diabetes have been constantly developed and improved, and maternal and infant outcomes of gestational diabetes patients have been greatly reduced. Standardized screening treatment has significantly reduced the incidence of maternal and infant complications of gestational diabetes mellitus. Therefore, it is very important to preform timely and accurate screening for patients with gestational diabetes.  **How much are you willing to pay for this screening** | | |
| --- | --- | --- |
| **Screening ways** | **Advantage** | **Disadvantage** |
| **75g（one-step）** | 1. Pregnant women with mildly high blood glucose may also be diagnosed as gestational diabetes and receive relative treatment. 2. You only need to come to the hospital once. | 1. Relatively high times of blood draws;  2. The waiting time is relatively long;  3. It may put more burden on pregnant women and society. |
| **50g（two-step）** | 1. Relatively fewer times of blood draws;  2. The waiting time is relatively short;  3. It is not easy to cause excessive screening. | 1．May cause missed diagnosis  2. May need to come to the hospital twice. |

| **75gOGTT**  **（one-step）** | | **50gGCT**  **（two-step）** | |
| --- | --- | --- | --- |
| WTP Bid Value |  | WTP Bid Value |  |
| Protest-O |  | Protest-O |  |
| Ture-0 |  | Ture-0 |  |
| 5 |  | 5 |  |
| 10 |  | 10 |  |
| 20 |  | 20 |  |
| 30 |  | 30 |  |
| 40 |  | 40 |  |
| 50 |  | 50 |  |
| 60 |  | 60 |  |
| 70 |  | 70 |  |
| 80 |  | 80 |  |
| 90 |  | 90 |  |
| 100 |  | 100 |  |
| 120 |  | 120 |  |
| 140 |  | 140 |  |
| 160 |  | 160 |  |
| 180 |  | 180 |  |
| 200 |  | 200 |  |

1. **Maternal preferred choice for “one step” and “two step” strategies**

| question | If there are two screening methods available (advantages and disadvantages of the two methods are shown below):   1. One step:75gOGTT 2. Two step: 50gGCT   which one do you prefer | | |  |
| --- | --- | --- | --- | --- |
| **Screening ways** | | **Advantage** | **Disadvantage** | |
| **75g（one-step）** | | 1. Pregnant women with mildly high blood glucose may also be diagnosed as gestational diabetes and receive relative treatment.  2. You only need to come to the hospital once. | 1. Relatively high times of blood draws;  2. The waiting time is relatively long;  3. It may put more burden on pregnant women and society. | |
| **50g（two-step）** | | 1. Relatively fewer times of blood draws;  2. The waiting time is relatively short;  3. It is not easy to cause excessive screening. | 1.May cause missed diagnosis  2. May need to come to the hospital twice. | |
